# Mother–Infant Interaction and Maternal Postnatal Psychological Distress Associate with Child’s Social-Emotional Development During Early Childhood: A FinnBrain Birth Cohort Study

**DOI:** 10.1007/s10578-024-01694-2

**Published:** 2024-04-16

**Authors:** Hetti Lahtela, Marjo Flykt, Saara Nolvi, Eeva-Leena Kataja, Eeva Eskola, Katja Tervahartiala, Juho Pelto, Alice S. Carter, Hasse Karlsson, Linnea Karlsson, Riikka Korja

**Affiliations:** 1 FinnBrain Birth Cohort Study, Department of Clinical Medicine, Turku Brain and Mind Center, University of Turku, Turku, Finland; 2 Department of Psychology, University of Turku, Turku, Finland; 3 Turku Institute for Advanced Studies, University of Turku, Turku, Finland; 4 Department of Psychology and Logopedics, Faculty of Medicine, University of Helsinki, Helsinki, Finland; 5 Department of Psychology, University of Massachusetts Boston, Boston, USA; 6 Centre for Population Health Research, Turku University Hospital and University of Turku, Turku, Finland; 7 Department of Psychology, University of Jyväskylä, Jyvaskyla, Finland; 8 Expert Services, Turku University Hospital, Turku, Finland; 9 Department of Psychiatry, University of Turku and Turku University Hospital, Turku, Finland; 10 Department of Pediatrics, Turku University Hospital and University of Turku, Turku, Finland; 11 Faculty of Social Sciences (Psychology), Tampere University, Tampere, Finland; 12 Department of Mathematics and Statistics, University of Turku, Turku, Finland

**Keywords:** Mother–infant interaction, Maternal psychological distress, Child social-emotional development, Social competence

## Abstract

We studied the effects of mother–infant interaction and maternal pre- and postnatal psychological distress on children’s social-emotional problems and competences, as well as whether interaction quality moderates the association between distress and children’s outcomes. Maternal pre- and postnatal psychological distress were measured using the SCL and EPDS questionnaires, whereas mother–infant interaction was measured when the child was 8 months old using the EA Scales. Children’s social-emotional development was measured using the BITSEA questionnaire at 2 years old and using the SDQ questionnaire at 4 years old, where higher maternal structuring was associated with fewer social-emotional problems in children and higher maternal sensitivity was associated with greater social-emotional competence in children at 2 years old. Further, higher postnatal distress was found associated with greater social-emotional problems at 2 years old, though neither these effects nor moderating effects at 4 years old were observed after multiple-comparison corrections. Our findings support direct associations of both mother–infant interaction and maternal postnatal psychological distress with children’s social-emotional development during toddlerhood.

**Supplementary Information:**

The online version contains supplementary material available at 10.1007/s10578-024-01694-2.

## Introduction

The quality of mother–infant interaction contributes to various aspects of children’s development, especially socially and emotionally [1–3]. Thus, social-emotional development issues typically present as behavioral (externalizing) and emotional (internalizing) symptoms [4], where the former are characterized by emotional and behavioral lability and may include aggressive and outward-directed behaviors, such as conduct problems, impulsiveness, and hyperactive behavior [5]. Meanwhile, the latter symptoms include anxiety, depression, and pronounced withdrawal [5]. Social-emotional competence is a key aspect of children’s development, reflecting adaptive social-emotional abilities; thus, a child with good social-emotional competence is better able to form and maintain developmentally adequate relationships with peers and adults [6, 7]. Social-emotional problems in early childhood predict later problems in academic functioning and peer relationships [8, 9]. In fact, the estimated prevalence of social-emotional problems in toddlers (2 years old) and preschoolers (4 years old) varies from 7 to 25% [8, 10], placing up to one in every four children at risk for social-emotional problems.

In addition, maternal depression and anxiety symptoms, that is, maternal psychological distress, are prevalent risk factors of compromised social-emotional development in children [11]. For example, accumulating evidence shows that maternal pre- and early postnatal psychological distress may contribute to later social-emotional problems in children [12, 13]. Yet, the contributions of different aspects of mother–infant interaction to the domains of children’s social-emotional development across early childhood in the context of maternal pre- and postnatal distress are not well understood. In addition, the results are still inconclusive regarding the moderating role of mother–infant interaction in the associations between maternal pre- and postnatal distress and children’s social-emotional development [14, 15]. According to some studies, high-quality mother–infant interaction may protect children’s social-emotional development from the negative consequences of maternal postnatal psychological distress [16, 17], but other studies have failed to identify any moderating effects [14]. Thus, a more nuanced understanding of the independent contributions of maternal pre- and postnatal distress, as well as of the various contributions of mother–infant interaction to children’s social-emotional development, is needed.

## Mother–Infant Interaction and Children’s Social-Emotional Development

Children’s early development takes places within the context of mother–infant dyads, where infants are primarily dependent on the external co-regulation of emotions and behavior provided by caregivers [18–20]. Thus, a key aspect of mother–infant interaction behavior is maternal sensitivity, where insensitive interactions signify a compromised capacity to read an infant’s emotional cues and hence to support the infant’s regulatory efforts adequately [20–22]. Mothers with higher sensitivity are thus better able to co-regulate their infants, leading to fewer social-emotional problems, such as externalizing symptoms [23, 24], and better social-emotional competence [25] later in their development. Maternal sensitivity has also demonstrated persistent associations with children’s social competence, even until adolescence and early adulthood [26]. Alongside sensitivity, other aspects of mother–infant interaction may affect children’s social-emotional development. For example, maternal strategies of structuring/scaffolding, such as distracting the child from a prohibited stimulus and using reasoning statements, enhance emotional self-regulation and social-emotional competence, such as prosocial behavior in toddlerhood and preschool [25, 27–29]. In addition, intrusiveness, which refers to an overcontrolling parental style, has been negatively associated with children’s social-emotional development, leading to greater negative emotionality in children. Finally, parental hostility, that is, harsh and negative behavior toward a child, has been found associated with more externalizing-type peer and relationship problems in early childhood [30]. In their review [31] Schneider et al. summarized the literature, arguing that parenting quality affects the development of children’s social-emotional problems in terms of both behavioral and emotional symptoms from infancy to 4 years old.

## Maternal Pre- and Postnatal Psychological Distress and Children’s Social-Emotional Development

Rogers et al. [32] concluded that both maternal pre- and postnatal psychological distress are associated with children’s long-term social-emotional development beyond infancy, with moderate effect sizes. That is, prenatal psychological distress has been found associated with the precursors of children’s social-emotional problems in infancy, such as greater negative emotionality [11] and behavioral and emotional symptoms in toddlerhood [33], middle childhood [34], and adolescence [32, 35]. Postnatally, maternal psychological distress affects children’s social-emotional development, leading to poorer emotion regulation and less social engagement in infancy [36, 37], increased behavioral symptoms during toddlerhood [38] lower social competence in middle childhood [39], lesser overall social-emotional development from infancy to middle childhood [32], and emotional symptoms even in adolescence [40, 41].

Both maternal pre- and postnatal distress are thus known to affect child development and behavior, but the routes may differ partially. Genetic factors influence the emergence of children’s social-emotional problems, such as internalizing and externalizing due to both pre- and postnatal maternal distress [42]. According to prenatal programming theory, intrauterine exposure to elevated levels of such agents as stress hormones or inflammatory cytokines may alter the development of the fetus’ brain and stress systems, including the hypothalamic–pituitary–adrenocortical (HCA) axis, one of the major stress systems in the human body [43] These changes may consequently lead to altered emotional and behavioral development postnatally [44], and maternal prenatal distress might lead to altered neurocognitive and emotional adaptations during pregnancy [45], possibly resulting in lowered maternal sensitivity [45]. Postnatal distress can also lead to compromised mother–infant interaction, with potentially harmful effects on child development [46]. However, the prenatal and early postnatal phases (that is, the first 12 months) seem not to be the only periods that affect children’s social-emotional development [47]. In a fairly recent study [47], maternal psychological distress in toddlerhood was found associated most strongly with children’s social-emotional outcomes, including externalizing and internalizing symptoms and social competence at age 5, when the effects of pre- and early postnatal distress were considered. Hence, it is important to control also for the level of concurrent maternal psychological distress when assessing the role of early maternal distress and different aspects of mother–infant interaction on children’s social-emotional development. Yet, an improvement of our understanding of the different roles of mother–infant interaction in conjunction with maternal pre- and postnatal psychological distress is still needed [15]. Hence, our aim was to use a prospective study design to fill this gap in the literature.

## Mother–Infant Interaction, Psychological Distress, and Social-Emotional Development

Although an association between early maternal psychological distress and children’s social-emotional developmental outcomes has been documented, the effect sizes have ranged from moderate to small [32, 48]. Hence, it is argued that some moderating factors play a role in determining the magnitude of these associations [49]. Research has demonstrated that the quality of mother–infant interaction is one of the strongest potential candidates for buffering the harmful impacts of maternal psychopathology on children’s social-emotional development postnatally [1, 17, 50]. Wurster et al. [17] demonstrated that the association between parents’ adverse childhood experiences and parental mental health on children’s social-emotional problems remained significant only among children whose parents demonstrated low emotional availability (EA) in caregiver–child interactions. In Mäntymaa et al.’s [50] study, parental distress had a greater impact on children’s behavioral and emotional symptoms when the quality of parent–child interactions was compromised, whereas another study showed that maternal depressive symptoms were associated with children’s depressive symptoms only among children with insecure attachments [16]. Emotional availability has also shown moderation effects in associations between maternal prenatal psychological distress and young children’s externalizing and internalizing symptoms [49, 51] and infant mental development [52]. Finally, Bergman and colleagues [53] found that mother–infant interaction buffered the impact of maternal prenatal distress on infant temperament and fearfulness. Earlier studies provide stronger evidence of a “buffering” effect (vs. mediating effect) of higher quality mother–infant interaction from the possibly harmful effects of the maternal prenatal psychological distress on the child’s social-emotional development [49, 51, 52]. However, studies on this topic are scarce, and not all show unequivocal findings. For example, Korja and McMahon [14] found no moderating effects of maternal emotional availability on the association between prenatal distress and emotional negativity in children; thus, more knowledge is needed about the possible moderating effects of different aspects of mother–infant interaction on children’s social-emotional development.

## Present Study

Ample evidence exists demonstrating that mother–infant interaction, as well as maternal psychological distress, both pre- and postnatally, are associated with children’s social-emotional development. Yet, less is known about the contributions of different aspects of mother–infant interaction to children’s social-emotional development, especially when the effects of both maternal pre- and postnatal psychological distress are considered [47, 54]. As such, the moderating role of mother–child interaction in the associations between prenatal psychological distress and children’s social-emotional development must be investigated further [14, 15, 53].

In this study, we investigated the effect of the association between the quality of mother–infant interactions when the child was 8 months of age and prenatal (at 14, 24, and 34 gestational weeks) and postnatal (when the child is 3, 6, and 12 months old) psychological distress among mothers on the different aspects of child social-emotional development at 2 and 4 years old. First, we investigated whether mother–infant interaction (sensitivity, structuring, non-intrusiveness, and non-hostility) was associated with children’s social-emotional development at 2 and 4 years old when the effects of maternal psychological distress were considered. Next, we examined whether maternal pre- and postnatal distress were associated with children’s social-emotional development at 2 and 4 years old, when the effects of mother–infant interaction were controlled. Children’s social-emotional development was measured as the total number of social-emotional problems and total level of social-emotional competence at 2 years old and as externalizing and internalizing symptoms and social competence at 4 years old. We hypothesized that (a) higher-quality mother–infant interaction in all areas would be associated with fewer social-emotional problems and higher social-emotional competence at 2 and 4 years old and that (b) higher maternal distress pre- and postnatally is associated with more social-emotional problems and lower social-emotional competence at 2 and 4 years old, and current maternal distress was controlled for in all models.

Third, we investigated whether mother–infant interaction quality moderates the association between maternal prenatal distress and children’s social-emotional problems and social-emotional competence. Based on the prior literature, we hypothesized that (c) higher levels of maternal sensitivity, structuring, non-intrusiveness, and non-hostility would diminish the adverse effects of maternal psychological distress on children’s social-emotional problems and social-emotional competence. Further, multiple comparison corrections were made to the regression models.

## Methods

### Participants and Procedures

The sample was drawn from families participating in the FinnBrain Birth Cohort Study [55], a prospective study designed to investigate the effects of early life stress on child development. The study population initially comprised pregnant women recruited during their first-trimester ultrasound (GA = 12 weeks) between December 2011 and April 2015 by a research nurse (n = 3838 families), and the subpopulation in this study consisted primarily of a case–control population, i.e., the Focus Cohort, drawn from the main cohort, comprising mothers who self-reported high levels of prenatal anxiety or depression symptoms (highest 25th percentile/cases) versus lower levels of prenatal anxiety and depression symptoms (lowest 25th percentile/controls) and their infants (N = 1227). The protocol was followed to ensure a sufficient variation in symptom scores.

**Fig. 1:**
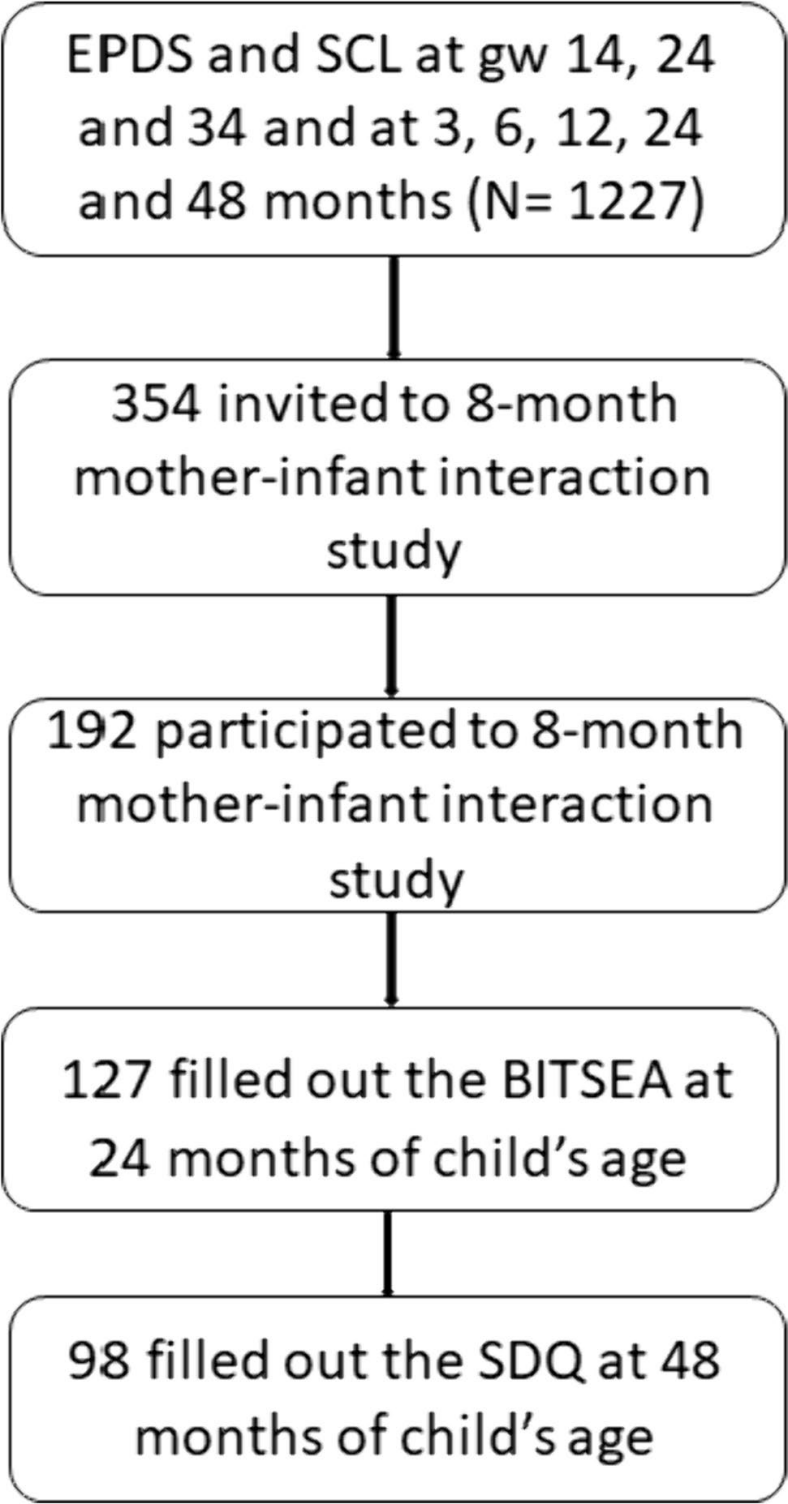
Flowchart of the sample

Focus Cohort families enriched by other actively participating cohort families were invited to participate in Child Development and Parental Functioning Lab study visits when the child was 8 months old. Mother–infant interaction was added later to the study protocol, leading to a smaller sample. For the 8 month study visit, 354 families were invited for the observational mother–infant interaction assessment, 192 of whom participated (55.1%). The same mothers were invited to complete questionnaires concerning their children’s social-emotional development at 2 and 4 years old, where 127 and 98 mothers agreed to participate at 2 years (35.9%) and 4 years (27.7%) postpartum, respectively, as part of this large birth cohort study. Thus, the final samples used in the analyses were N = 127 mothers and their 2-year-old children and N = 98 mothers and their 4-year-old children, respectively (Fig. 1). The mothers who participated in the 8 month study visit and who completed the child social-emotional development questionnaire at 2 years had a higher income (χ^2^ = 0.02) and higher education level (χ^2^ = 0.00) than those who dropped out after the 8 month study visit. Further, the mothers who had participated in the 8 month study visit and completed the social-emotional development questionnaire at 4 years demonstrated a greater likelihood of being primiparous (χ^2^ = 0.03), a higher education level, and a higher income (χ^2^ = 0.03) than the mothers who dropped out after the 8 month study visit. Depressive and anxiety symptom questionnaires were also completed at 14, 24, and 34 weeks’ gestation and at 3, 6, 12, 24, and 48 months postpartum (excluding the anxiety questionnaire at 12 months). As well, participants gave their written consent, data were analyzed according to data privacy guidelines, and the Joint Ethics Committee of the University of Turku and the South-Western Hospital District approved the study protocol.

## Measures

### Background Information

The background data used in this study were collected over the pre- and postnatal periods as follows: mothers completed a background information questionnaire at 14 weeks’ gestation that collected information on education level and monthly income, among other data, whereas information on maternal age and child sex was obtained from national birth registries [55].

### Maternal Pre- and Postnatal Distress

Depressive symptoms were measured using the Edinburgh Postnatal Depression Scale (EPDS), a widely used self-report featuring 10 items, each of which has four possible responses ranging from 0 to 3 for minimum and maximum total scores of 0 and 30, respectively [56]. The EPDS has been proven a valid measure postnatally, as well as prenatally [57]; as such, the total sum of scores was used as a continuous variable, where internal consistency was adequate, with a Cronbach’s alpha value ranging between 0.85 and 0.91.

Anxiety symptoms were measured using the anxiety subscale from the Symptom Checklist-90-Revised (SCL-90-R®), which has been proven a valid questionnaire to measure psychological symptoms, including anxiety, depression, and obsessive–compulsive disorder [58, 59]. A sum of the total scores was used as a continuous variable, and the internal consistency of the SCL-90-R® was adequate, with a Cronbach’s alpha value ranging between 0.83 and 0.91.

As the primary aim was to assess maternal psychological distress at different time points when studying the effects of mother–infant interaction on children’s social-emotional development, a sum score of combined anxiety and depressive symptoms prenatally versus a combined score in the early postnatal period was used in the analyses. The combined score for prenatal distress was determined by first calculating the EPDS and SCL-90-R® means at 14, 24, and 34 weeks’ gestation, then by standardizing and summing them, and finally by standardizing the sum to obtain an interpretable value. The postnatal score was calculated in a corresponding manner by combining the symptom scores at 3 and 6 months (and depressive symptom scores at 12 months, except anxiety scores). Concurrent symptoms were measured at 2 and 4 years, and they covaried, a result parallel to the measurements of children’s social-emotional outcomes. Finally, each sum score of the current symptoms of depression and anxiety was used at each specific age point.

### Mother–Infant Interaction

Mother–infant interaction was coded from a 20-min free-play session, where the mothers and infants were offered age-appropriate toys and instructed to play as they would normally, regardless of whether the mothers actually used the toys. The videotaped mother–infant interaction data were coded using the Emotional Availability Scales (EA Scales; [60]), which consist of four adult dimensions and two child dimensions to describe interactions between an adult and a child. These include adult sensitivity, structuring, non-intrusiveness, non-hostility and child responsiveness, and adult involvement [60]. In this study, only the adult scales were used to evaluate the quality of mother–child interaction, as the effects of parenting on children’s social-emotional outcomes was the focus of interest. Also, we wanted to keep the amount of the statistical models as low as possible, hence the child subscales were left out also for statistical reasons. Sensitivity is defined as the parent’s ability to interact with the child in a positive and genuine way and to respond to the child’s emotional cues while maintaining a healthy emotional connection within the dyad. Meanwhile, structuring refers to the adult’s ability to support situations in a manner that is age appropriate and well-received by the child. Further, non-intrusiveness refers to the adult’s ability to give the child an age-appropriate amount of space, to respect the child’s independence, and to respond to the child’s initiatives during play. Finally, non-hostility means the adult acts peacefully toward the child by avoiding passive aggression, i.e., a sarcastic or demeaning style, or overt aggression [60]. In the fourth edition of the EA Scales, a direct/global score between 1 and 7 is given for each scale, reflecting the evaluator’s overall view of the interactions and emotional relationship within the dyad. A score between 1 and 2–2.5 for all six scales is considered highly problematic, a score between 3 and 3.5 indicates detachment in the relationship, and a score between 4 and 5 indicates complicated EA. In turn, scores ranging between 5.5 and 7 are indicative of dyadic, healthy EA in the relationship between mother and child [61]. Interaction data were coded by two blinded and trained coders with confirmed reliability, as recommended in the EA guidelines, within Biringen’s laboratory. The intra-class correlations at the 8-month measurement point were 0.80 for sensitivity, 0.72 for structuring, 0.85 for non-intrusiveness, and 0.70 for non-hostility, and the scales were used as continuous variables.

### Social-Emotional Problems and Social-Emotional Competence at 2 Years Old

Children’s social-emotional development at 2 years old was measured using the Brief Infant–Toddler Social and Emotional Assessment (BITSEA), completed by the mothers. The BITSEA is a valid and reliable caregiver questionnaire for screening toddlers’ social-emotional problems and social-emotional competence [4] in both normative and clinical samples. It can be used with children aged 12 to 36 months, and it consists of 42 items. Each question is scored on a scale from 0 to 2, with 0 meaning “Not true/rarely,” 1 meaning “Somewhat true/Sometimes,” and 2 meaning “Very true/Often.” The BITSEA Problem Scale comprises 31 items, with scores ranging from 0 to 62, whereas the BITSEA Competence Scale includes 11 items, with scores ranging from 0 to 22. In this study, the total problem and social competence scale scores were used as proxies of social-emotional development at 2 years old, where the former measures social-emotional problems, such as aggression, defiance, overactivity, shyness, anxiety, and social withdrawal and the latter measures such aspects as compliance, mastery motivation, prosocial behavior, and empathy. In the present study, the sum of total scores was used as a continuous variable, and the total score indicates the social-emotional problems. This was done to keep the number of models as low as possible, as symptoms at this age point are less differentiated than at preschool age. The Cronbach’s alpha values were 0.59 for the Competence Scale and 0.72 for the Total Problem Scale, which is in line with another study that evaluated social-emotional development in toddlers [9].

### Social-Emotional Problems and Social Competence at 4 Years Old

Social-emotional development was measured at 4 years old using the Strengths and Difficulties Questionnaire (SDQ), a valid and reliable tool used to assess the social-emotional adjustment of children and youth aged 3–16 years [62, 63]. The questionnaire, completed by the mothers, consists of five subscales, each including five items, that measure emotional symptoms, conduct problems, hyperactivity-inattention, peer problems, and prosocial behavior. Each question was scored on a Likert scale of 0–2, with answers representing how well each attribute applies to the child. The subscales (excluding the prosocial behavior subscale) were grouped into two scales: internalizing symptoms, combining the Emotional Symptoms and Peer Problems subscales, and externalizing symptoms, combining the Conduct Problems and Hyperactivity/Inattention subscales, as recommended by the community samples [64]. The scores of the externalizing and internalizing subscales range from 0 to 20, and the Prosocial behavior subscale scores range from 0 to 10. The outcome scales in the current study were the externalizing subscale, internalizing subscale, and prosocial behavior score, which indicates social competence, and the Cronbach’s alpha values for the subscales were as follows: Emotional symptoms 0.647 and peer problems: 0.561 which together form the internalizing scale. Cronbach’s alphas for conduct problems subscale was 0.728 and hyperactivity-inattention subscale 0.726 which together form the externalizing scale and 0.730 for the prosocial behavior subscale, respectively.

### Statistical Analyses

IBM SPSS Statistics (Version 26) and R (Version 3.6.3) were used for data analyses. First, correlations between maternal EA variables (sensitivity, structuring, non-intrusiveness, and non-hostility), maternal pre- and postnatal distress, and the outcome variables of the total social-emotional problems and social-emotional competence (at 2 years old) and externalizing symptoms, internalizing symptoms, and social competence (at 4 years old) were calculated. In addition, the associations between the background and outcome variables were studied using Pearson’s correlation or one-way analysis of variance. Next, multiple linear regression models were used to investigate the associations between the study and outcome variables, and they examined the associations among mother–infant interaction quality at 8 months; maternal psychological distress prenatally, postnatally, and concurrently (i.e., at 2 and 4 years old, as a covariate); and children’s social-emotional problems and social-emotional competence at 2 and 4 years old. Each model included one EA scale (4x), pre- and postnatal distress scales, and the relevant covariates of concurrent distress (at either 2 or 4 years old), child sex, and one of the outcome variables (2 at the 2-year and 3 at the 4-year mark). Child sex was selected as a covariate because it was the only background variable associated with child social-emotional problems in our sample. Second, we formed linear regression analyses to investigate the possible moderating effects of mothers’ emotional availability on the associations between maternal prenatal distress and children’s social-emotional problems and social-emotional competence at 2 and 4 years old. Due to multiple tests performed, the *p* values related to the study questions were adjusted using the Benjamini–Hochberg method [65], and adjustments were made separately for each hypothesis concerning the regression models.

## Results

### Descriptive Statistics

The demographic characteristics of the sample are presented in Table 1. Child sex was associated with social-emotional competence at 2 years (*p* = 0.009) and 4 years old (*p* = 0.028), and girls were perceived as more competent than boys by their mothers. Further, none of the other background variables was associated with social-emotional outcomes at 2 and 4 years old.

**Table 1:** Demographic characteristics of the study population

Characteristic	*N* = 127 (at 2 years)	*N* = 94 (at 4 years)
Maternal age at child birth, mean (*SD*)	31.5 (4.0)	31.7 (4.1)
Maternal education in pregnancy (%)		
Low^a^	25 (17.1)	15 (15.6)
Middle^b^	46 (37.4)	36 (37.5)
High^c^	56 (45.5)	45 (46.9)
Infant sex, male (%)	61 (48.8)	49 (50)
Maternal monthly net income in pregnancy (%)^d^		
< 1500	33 (26.8)	24 (25.0)
1501–2500	76 (61.8)	61 (63.5)
2501–3500	13 (10.6)	9 (9.4)
> 3500	1 (0.8)	2 (2.1)
EA (mean, SD)		
Sensitivity	5.3 (1.4)	5.3 (1.2)
Structuring	5.1 (1.5)	5.1 (1.5)
Non-intrusiveness	5.8 (1.3)	5.7 (1.4)
Non-hostility	6.2 (1.0)	6.2 (1.0)
EPDS* (mean, SD)		
14 GW	4.6 (4.3)	4.2 (3.8)
24 GW	4.6 (4.5)	4.1 (4.0)
34 GW	4.5 (4.6)	4.2 (4.2)
3 months	4.0 (3.9)	3.9 (3.7)
6 months	4.5 (4.7)	4.3 (4.3)
1 year	5.0 (4.6)	4.8 (4.6)
2 years	4.8 (4.5)	4.6 (4.4)
4 years	4.1 (4.1)	4.3 (4.2)
SCL-90** (mean, SD)		
14 GW	3.1 (4.3)	2.6 (3.4)
24 GW	3.7 (4.9)	3.3 (4.5)
34 GW	3.2 (4.6)	2.8 (4.1)
3 months	2.6 (3.8)	2.5 (3.7)
6 months	3.0 (4.6)	2.8 (4.4)
2 years	3.3 (4.3)	3.0 (4.1)
4 years	3.1 (4.6)	3.3 (4.6)

### Bivariate Correlations Among Independent Variables

Next, we investigated the correlations among the predictor variables, and the results are shown in Table 2. All EA Scales significantly correlated with each other (r = 0.340–0.703): non-intrusiveness correlated with maternal psychological distress during pregnancy and when the child was 2 and 4 years old (r = − 0.192–-0.091). The mothers who demonstrated greater non-intrusiveness reported fewer distress symptoms at these time points. Further, sensitivity correlated with postnatal distress at 2 years postpartum (r = − 0.176), whereas prenatal distress correlated positively with postnatal distress at every timepoint (r = 0.699–0.798).

**Table 2:** Pearson correlations between the EA scales and maternal pre- and postnatal psychological distress (Non-corrected)

Variable	1	2	3	4	5	6	7	8
Sensitivity	–							
Structuring	.703***	–						
Nonintrusivenesss	.498***	.340***	–					
Nonhostility	.624***	.413***	.520***	–				
Prenatal distress	− .078	− .022	− .163*	− .045	–			
Postnatal distress during first year	− .075	− .065	− .091	.012	.752***	–		
Maternall distress at 2 years	− .176*	− .132	− .195**	− .101	.699***	.798***	–	
Maternal distress at 4 years	− .126	− .054	− .192**	− .010	.666***	.761***	.782***	–

*p < .05. **p < .01

### Mother–Infant Interaction, Maternal Psychological Distress, and Children’s Social-Emotional Problems

Next, regression models were created for each association among mother–infant interaction (sensitivity, structuring, non-intrusiveness, and non-hostility, respectively); maternal pre-, post-, and concurrent distress; and children’s social-emotional problems or social-emotional competence and the relevant covariates, the results of which are shown in Table 3. Only the models in which mother–infant interaction was initially statistically significantly associated with children’s social-emotional problems or social-emotional competence at either 2 or 4 years old are displayed, and non-significant associations are displayed in the supplementary material. Finally, multiple-comparison corrections were applied to each regression model.

**Table 3:** Linear regression model for the effects of maternal sensitivity and structuring on child’s social-emotional problems at 2 years

Variable	*B*	*SE*	*p*	CI 95%	Partial ETA^2^	*adj p*^a^
Sensitivity						
Sex (girl)	− 0.12	0.15	.445	[− 0.42; 0.19]	0.00	
Prenatal distress	− 0.01	0.11	.938	[− 0.23¸0.21]	5.13	0.96
Postnatal distress	0.51	0.15	.001**	[0.22; 0.80]	0.09	0.005
Current distress	0.03	0.13	.818	[− 0.23; 0.29]	0.00	
Sensitivity 8 months	− 0.15	0.08	.045*	[− 0.30; − 0.00]	0.33	0.107
Structuring						
Sex (girl)	− 0.09	0.15	.545	[− 0.39; 0.21]	0.00	
Prenatal distress	0.01	0.11	.898	[− 0.21¸0.24]	0.00	0.963
Postnatal distress	0.50	0.14	< .001**	[0.21; 0.78]	0.09	0.005
Current distress	0.03	0.13	.837	[− 0.23; 0.28]	0.00	
Structuring 8 months	− 0.20	0.07	.007**	[− 0.35; − 0.06]	0.06	0.042

*p < .05, **p < .01, ^a^Corrected *p* with the Benjamini–Hochberg method

Higher maternal sensitivity when the child was 8 months old was associated with fewer social-emotional problems at 2 years old in the model. In addition, higher maternal postnatal psychological distress (but not prenatal distress) was associated with more child social-emotional problems. However, after multiple-comparison corrections, this association did not remain statistically significant. In addition, higher maternal structuring at 8 months was associated with fewer social-emotional problems at 2 years old. Again, maternal postnatal psychological distress (but not prenatal distress) was associated with more social-emotional problems in children, but no other associations among study variables were identified regarding social-emotional problems at 2 years old.

Higher maternal sensitivity at 8 months postpartum was associated as well with fewer externalizing problems in children at 4 years old (Table 4) after accounting for child sex and maternal pre- and postnatal distress. However, this association did not remain significant after multiple-comparison corrections, and neither pre- nor postnatal psychological distress, nor other variables of mother–infant interaction, were found associated with externalizing or other social-emotional problems in children at 4 years old.

**Table 4:** Linear regression model for the effects of maternal sensitivity on child’s externalizing symptoms at 4 years

Variable	*B*	*SE*	*p*	CI 95%	Partial ETA^2^	*adj p *^*a*^
Sex (girl)	− 0.11	0.18	.540	[− 0.48; 0.25]	0.00	
Prenatal distress	0.05	0.17	.766	[− 0.28¸0.38]	0.00	0.928
Postnatal distress	0.23	0.16	.157	[− 0.09; 0.56]	0.02	0.377
Current distress	0.20	0.13	.124	[− 0.05; 0.46]	0.03	
Sensitivity at 8 months	− 0.21	0.10	.036*	[− 0.41; − 0.01]	0.05	0.434

*p < .05, **p < .01, ^a^Corrected *p* with the Benjamini–Hochberg method

Concerning children’s social-emotional competence, higher maternal sensitivity at 8 months was associated with improved social-emotional competence in children at 2 years old, after controlling for child sex and maternal pre- and postnatal distress (Table 5). Meanwhile, neither pre- nor postnatal psychological distress, nor other variables of mother–infant interaction, was found to be associated with children’s social-emotional competence at 2 or 4 years old.

**Table 5:** Linear regression model for the effects of maternal sensitivity on child’s social competence at 2 years

Variable	*B*	*SE*	*p*	CI 95%	Partial ETA^2^	*adj p*
Sex (girl)	0.33	0.16	.042*	[0.01; 0.66]	0.03	
Prenatal distress	− 0.12	0.12	.312	[− 0.36¸0.12]	0.01	0.500
Postnatal distress	− 0.22	0.15	.156	[− 0.52; 0.08]	0.02	0.380
Current distress	− 0.05	0.14	.723	[− 0.32; 0.22]	0.00	
Sensitivity at 8 months	0.21	0.08	.011*	[0.05; 0.36]	0.05	0.042

*p < .05, **p < .01, ^a^Corrected *p* value

In summary, after multiple-comparison corrections, only the associations between maternal structuring and reduced social-emotional problems in children, as well as between maternal sensitivity and improved social-emotional competence in children at 2 years old remained significant.

### Moderating Effects of Mother–Infant Interaction on Associations Between Maternal Prenatal Distress and Child Social-Emotional Development

The results concerning whether mother–infant interaction moderates the association between prenatal distress and child outcomes at 2 and 4 years old are presented in Table 6. In the initial models, maternal sensitivity moderated the association between prenatal distress and children’s internalizing and externalizing symptoms at 4 years old. Meanwhile, greater maternal sensitivity appeared to attenuate the association between maternal prenatal distress symptoms and children’s internalizing and externalizing symptoms at 4 years old. Maternal structuring also moderated the association between maternal prenatal distress and children’s internalizing symptoms at 4 years old, where the more structured the mother, the weaker the positive association between maternal prenatal distress and internalizing symptoms in children at 4 years old. In addition, maternal non-hostility moderated the association between maternal prenatal distress and children’s internalizing symptoms at 4 years old. Conversely, the less hostile the mother, the weaker the positive association between maternal prenatal distress and internalizing symptoms in children at 4 years old. However, after multiple-comparison corrections, none of the moderation analyses remained statistically significant, and no other moderation effects were detected.

**Table 6:** Interaction effects of sensitivity, structuring and non-hostility on the associations between maternal prenatal psychological distress and child’s internalizing and externalizing symptoms at 4 years

Variable	*B*	*SE*	*p*	*CI* 95%	Partial Eta^2^	*adj. p*
Sensitivity on internalizing						
Sex (girl)	− 0.01	0.18	.972	[− 0.35, 0.36]	1.35	
Prenatal distress	0.20	0.17	.243	[− 0.14, 0.53]	0.01	
Postnatal distress	0.07	0.16	.671	[− 0.25, 0.39]	0.00	
Current distress	0.29	0.13	.023*	[0.04, 0.54]	0.06	
Sensitivity × prenatal distress	− 0.22	0.11	.049*	[− 0.45, − 0.00]	0.04	.596
Structuring on internalizing						
Sex (girl)	0.00	0.18	.989	[− 0.36, 0.37]	2.16	
Prenatal distress	0.14	0.18	.442	[− 0.21, 0.49]	0.01	
Postnatal distress	0.10	0.16	.529	[− 0.22, 0.43]	0.00	
Current distress	0.31	0.13	.016*	[0.06, 0.56]	0.06	
Structuring × prenatal distress	− 0.21	0.10	.047*	[− 0.41, − 0.00]	0.04	.647
Non-hostility on internalizing						
Sex (girl)	− 0.01	0.17	.941	[− 0.36, 0.34]	5.88	
Prenatal distress	0.04	0.19	.843	[− 034, 0.42]	0.00	
Postnatal distress	0.15	0.16	.378	[− 0.18, 0.47]	0.01	
Current distress	0.34	0.13	.001*	[0.09, 0.59]	0.07	
Hostility × prenatal distress	− 0.21	0.09	.020*	[− 0.38, − 0.03]	0.06	.647
Sensitivity on externalizing						
Sex (girl)	− 0.07	0.18	.700	[− 0.43, 0.29]	0.00	
Prenatal distress	− 0.04	0.17	.858	[− 037, 0.31]	0.00	
Postnatal distress	0.27	0.16	.098	[− 0.05, 0.59]	0.03	
Current distress	0.20	0.13	.124	[− 0.06, 0.45]	0.03	
Sensitivity × prenatal distress	− 0.23	0.11	.048*	[− 0.46, − 0.00]	0.04	.596

*p < .05

## Discussion

In this study, we investigated the effects of mother–infant interaction and maternal pre- and postnatal psychological distress on children’s social-emotional problems and social-emotional competence at 2 and 4 years old. In addition, we studied whether mother–infant interaction would moderate possible associations between prenatal distress and social-emotional problems or social-emotional competence in children at 2 and 4 years old.

We found that higher maternal structuring was associated with fewer social-emotional problems in children, whereas higher maternal sensitivity was associated with better social-emotional competence in children at 2 years old. Furthermore, maternal postnatal psychological distress, but not prenatal distress, was found to be independently associated with children’s social-emotional problems at 2 years old, and these findings remained significant even after multiple-comparison corrections. We did not identify any associations between mother–infant interaction or maternal psychological distress and the measurement outcomes in children at 4 years old, nor did we identify any moderating effects of mother–infant interaction that would have survived the multiple–comparison corrections.

Our finding concerning the association between higher maternal structuring during infancy and fewer social-emotional problems in children at 2 years old is in line with earlier studies showing that appropriate parental scaffolding, i.e., structuring, is associated with fewer social-emotional problems in children [23, 25]. From a developmental perspective, our finding is also plausible, as toddlerhood is a period of rapid development of their social, emotional, and cognitive abilities, during which time children must learn to navigate between external and internal emotion regulation [47, 66] after only just beginning to learn compliance and appropriate behaviors in different social settings. Our results support the importance of parental structuring, such as verbal explanations and limit setting in social situations, as children can then learn these skills, thus reducing social-emotional problems in early childhood. Through parental structuring, children learn different strategies for handling frustrating social situations [9] which in turn might reduce their externalizing symptoms. Accordingly, appropriate parental scaffolding has been shown to play an important role in promoting this development [9, 29].

Our second main finding, concerning the association between higher maternal sensitivity and children’s improved social-emotional competence in toddlerhood, is also in line with previous research demonstrating that maternal sensitivity promotes social-emotional competence in early childhood [23, 30]. In addition, higher maternal sensitivity early in a child’s life can lead to improved social-emotional competence in the child via regulatory patterns related to the attachment system [25]. For example, maternal warmth and appropriate responsiveness in interactions between mother and child, as well as the mother’s ability to soothe the infant in a state of distress, can support early emotional regulation in the infant and ensure a secure attachment style. Through sensitive mother–infant interaction, the infant forms internal working models that form the basis of a warm and sociable interaction style, allowing the child to rely on other people [67]. Consequently, a child who learns to regulate their emotions in the context of the parent–child relationship is also likely to employ these skills in future social interactions [29] an ability that may reflect better social-emotional competence. Contrary to our hypothesis and to earlier studies showing that negative caregiving behavior, such as hostility [30] or intrusiveness [68] associated with heightened levels of socio-emotional problems in children, we did not find any connection of non-hostility and non-intrusiveness with the different domains of children’s social-emotional development at either 2 or 4 years old. It might be that in our low-risk sample, the variation in the non-intrusiveness and non-hostility scales was insufficient to detect the effects of these maternal interactional domains on children.

The finding concerning the association between postnatal maternal psychological distress and children’s social-emotional problems is in line with earlier studies that have shown that postnatal depression and anxiety symptoms can affect the development of social-emotional functioning in children [32, 34]. That is, the mechanism through which postnatal distress may affect child well-being is thought to compromise mother–infant interaction, leading the mother to be less sensitive or less structured [32, 46] In our study, both mother–infant interaction and postnatal psychological distress were associated with toddler social-emotional development, but the mechanisms were not studied in greater detail.

Based on our results, the effects of mother–infant interaction and maternal psychological distress in the early postpartum period are associated more strongly with children’s social-emotional development than maternal psychological distress in the prenatal period. This is partly in line with earlier studies that have shown how maternal postnatal psychological distress predicts children’s social-emotional outcomes, including deficits in emotional regulation in infancy [36, 37] and externalizing symptoms during toddlerhood [38]. We did not find any associations between prenatal psychological distress and children’s social-emotional outcomes after the effects of postnatal distress and mother–child interaction were considered. This is contradictory to earlier findings, which have shown that prenatal anxiety and depressive symptoms are associated with child social-emotional outcomes [11, 33, 69]. One explanation could be that our sample was drawn from a general population with a relatively high educational background and relatively low maternal psychological distress levels. Recent studies also show that it is not pre- and postnatal distress alone, but also the chronicity of the distress across several periods that contributes to increased social-emotional problems and compromised social-emotional competence in children [38, 47, 70]. Our recent study from the same population with a larger sample showed that the trajectories of high-level persistent depression and anxiety symptoms that continued from pregnancy until 2 years postpartum had the strongest association with children’s outcomes [70]. The effects of distress in the pre- or postnatal period on children’s socio-emotional development were less consistent, as in our sample, concurrent maternal psychological distress was not associated with children’s outcomes, contrary to some earlier studies [47].

Based on our preliminary analyses, higher maternal sensitivity when the child was 8 months old was associated with fewer social-emotional problems in children at 2 years old and with fewer externalizing symptoms at 4 years old. In addition, in the initial analyses, higher maternal sensitivity, greater structuring, and non-hostility showed a trend toward moderating the association between maternal prenatal distress and children’s internalizing symptoms at 4 years old. In addition, maternal sensitivity showed a trend toward moderating the association between maternal prenatal distress and children’s externalizing symptoms at 4 years old. However, after multiple-comparison corrections, none of the moderating effects remained statistically significant, so they must be interpreted very cautiously. Still, the results were in the expected direction and might potentially indicate that higher maternal EA buffers the potentially harmful effects of maternal prenatal distress on children’s later social-emotional development at preschool age. For example, earlier studies have shown that higher sensitivity and a secure attachment style might diminish the association between maternal psychological distress and the different domains of child developmental problems, including social-emotional development [14, 49, 51, 53, 71]. One reason that we found only very limited evidence of the moderating effects of mother–infant interaction on the relation between maternal prenatal psychological distress and children’s social-emotional development might be that the moderating effects only occur at chronic or clinical levels of maternal distress, as some studies indicate [16, 71]. Another explanation is that the power of the data was insufficient to detect such associations; thus, further studies must be completed to clarify the buffering effects of mother–child interaction on maternal prenatal distress and children’s social-emotional development, perhaps using at-risk populations.

Our results demonstrated additional evidence of the role of mother–infant interaction in children’s social-emotional development during toddlerhood compared to the preschool years. As toddlerhood is a period of the rapid development of children’s social-emotional abilities [47, 66], it might be that the associations between mother–infant interaction and children’s outcomes are clearest in this phase. Because maternal sensitivity and structuring support children’s development of their emotion regulation in the context of the attachment relationship and mother–infant interaction [20, 21, 72], especially in early childhood [47, 66], it is plausible that maternal EA is also related to children’s social-emotional outcomes. Based on our results, it might be that the influence of early mother–infant interaction lessens by preschool age, at least in a low-risk population, such as our sample. This is understandable, as other factors alongside mother–infant interaction, such as out-of-home childcare and peer groups, have an enhanced impact on children’s development [26]. In addition, the importance of the other parent is increasingly important; further, the relationship between mother and child can change over time as the child ages, so it is understandable that associations diminish over time. However, our results concerning the associations between mother–infant interaction and children’s social-emotional outcomes at 4 years old are contrary to the findings from other studies having found that an early caregiving environment plays an important role in social-emotional development, including at preschool age and even until adulthood [26, 29]. It might be that cumulative risk factors, including socioeconomic factors, in addition to mother–child interaction, increase the likelihood of persistent effects on child social-emotional outcomes [26].

### Study Strengths and Limitations

The strength of our study is its longitudinal design and measurement of children’s social-emotional development at two different age points, in toddlerhood (2 years old) and at preschool age (4 years old). Another strength is that mother–infant interaction was assessed using observational methods, which are considered more objective than self-reports in measuring parenting [73]. Importantly, we were able to assess the effects of mother–infant interaction on children’s social-emotional development while controlling for both prenatal, as well as postnatal psychological distress, which has not been done in many previous studies [47, 54]. Still, there are several limitations to our study. First, children’s social-emotional development was only assessed using questionnaires, which may lead to a reporting bias, especially with the mothers being the only responders [74]. In addition, maternal psychological distress was assessed with self-reports, and the symptom scores were used as continuous variables, despite not reflecting a clinical diagnosis; accordingly, the results might differ in a clinical setting. A further limitation is that we were unable to study father–infant interaction, and the sample sizes and attrition were additional limitations, despite being difficult to avoid in a prospective study design. A final limitation was the fairly low internal consistency of the social-emotional questionnaires, BITSEA and SDQ, despite being in line with other studies of normative populations [9, 75]. Still, despite the fairly low alphas we chose to use these measurements, since they are widely used questionnaires and are recommended to use in their original form and have shown satisfactory and adequate reliability and validity in other normative samples [76–78]. One reason for the poor reliability scores for these questionnaires might be due to the fact that these scales are intended to be medical screeners and not to measure underlying concepts as much as the number of symptoms, resulting also in ordinal scales that may induce problems in consistency analysis.

### Clinical Implications

As children’s social-emotional development predicts their everyday psychosocial functioning [79] understanding the early factors impacting this development has several important clinical implications. The results support the view that sensitivity and structuring in mother–infant interaction might be useful targets in interventions to improve children’s social-emotional development. In addition, more resources should be targeted toward identifying both depression and anxiety symptoms in maternity clinics, as during pregnancy, they can be associated with mother–child interaction [80] and might predict later symptoms during the postnatal period [81], which, based on our results, might contribute to children’s social-emotional outcomes in early childhood, alongside mother–infant interaction. Because cumulative adversities place children at an even greater risk of future disturbances to their social-emotional development, interventions should be targeted, especially toward families with multiple risk factors [12, 13, 47].

In the future, more studies with prospective designs featuring at-risk populations are needed to study the role of mother–infant interaction in children’s long-term social-emotional development in the context of maternal pre- and postnatal psychological distress in different populations. More research is needed especially among older children, which would enhance the knowledge of the longitudinal associations between mother–infant interaction and children’s later social-emotional outcomes. This will aid in targeting different interventions to relevant populations. Also, studying the role of child’s early interaction characteristics and the possible mediating role of mother-infant interaction would be important in future studies as it has been shown that mother-infant interaction could also serve as a mediating factor in the associations between maternal psychological distress and child social-emotional problems particularly considering the postnatal maternal distress [82].

## Summary

Studies simultaneously examining the role of pre- and postnatal maternal psychological distress and mother-infant interaction on children’s early social-emotional development are scarce. Especially, the potential moderating role of mother-infant interaction on the associations between prenatal distress and children’s social-emotional outcomes is not well understood. Our study showed that mother-infant interaction, specifically higher maternal structuring, associated with less child social-emotional problems at 24 months. Similarly, higher sensitivity was associated with children’s better social competence at 24 months. Additionally, higher maternal postnatal psychological distress associated with children’s social-emotional problems, while prenatal distress did not associate with any of the child outcomes after taking into account the effects of mother-infant interaction and postnatal distress. We did not find evidence for moderating effects of mother-infant interaction on the associations between maternal prenatal distress and child social-emotional development. Our results indicate that lower mother-infant interaction quality and maternal postnatal psychological distress are negatively associated with toddlers’ social-emotional development. Our findings support the need for early interventions targeting both maternal perinatal psychological distress as well as mother-infant interaction in preventing possible social-emotional difficulties in young children.

## Supplementary Information

Below is the link to the electronic supplementary material.

Supplementary file1 (DOCX 46 kb)

## Data Availability

The datasets generated for the present study will not be made publicly available because of restrictions imposed by Finnish law and the study’s ethical permissions do not allow sharing of the data used in this study. Requests to access the datasets should be directed to the principal investigator of the FinnBrain Birth Cohort Study.
