# Supplementary material for: Mother–Infant Interaction and Maternal Postnatal Psychological Distress Associate with Child’s Social-Emotional Development During Early Childhood: A FinnBrain Birth Cohort Study
Source: Child Psychiatry Hum Dev. 2024 Apr 16;57(1):172–87. doi: 10.1007/s10578-024-01694-2 (PMC12971811; doi:10.1007/s10578-024-01694-2)
Supplement: Supplementary file 1 — Supplementary file1 (DOCX 46 kb) [file 10578_2024_1694_MOESM1_ESM.docx]

**Table 1.** *The associations between mother-infant interaction, maternal psychological distress and child social-emotional problems at 2 years.*

| Variable | *B* | *SE* | | *p* | *CI 95%* | *Partial ETA²* | | *adj p ^a^* | |
| --- | --- | --- | --- | --- | --- | --- | --- | --- | --- |
| **Non-intrusiveness**  Sex (girl)  Prenatal distress  Postnatal distress  Current distress  Non-intrusiveness 8 months | -0.13  -0.01  0.49  0.08  0.06 | 0.16  0.12  0.15  0.13  0.08 | | 0.42  0.96  0.00  0.53  0.46 | [-0.43:0.18]  [-0.23:0.22]  [0.19:0.78]  [-0.18:0.34]  [-0.10:0.21] | 0.00  1.84  0.08  0.00  0.00 | | 0.96  0.01 | |
| **Non-hostility**  Sex (girl)  Prenatal distress  Postnatal distress  Current distress  Non-hostility 8 months | -0.13  -0.02  0.51  0.06  -0.03 | | 0.16  0.11  0.15  0.13  0.08 | 0.39  0.87  0.00  0.64  0.68 | [-0.44:0.17]  [-0.25:0.21]  [0.21:0.80]  [-0.20:0.32]  [-0.18:0.12] | | 0.01  0.00  0.09  0.00  0.00 | | 0.96  0.00 |

**Table 2.** *The associations between mother-infant interaction, maternal psychological distress and child social-emotional competence at 2 years.*

| Variable | *B* | *SE* | | *p* | *CI 95%* | *Partial ETA²* | | *adj p ^a^* | |
| --- | --- | --- | --- | --- | --- | --- | --- | --- | --- |
| **Structuring**  Sex (girl)  Prenatal distress  Postnatal distress  Current distress  Structuring 8 months | 0.32  -0.13  -0.20  -0.07  0.16 | 0.17  0.12  0.16  0.14  0.08 | | 0.05  0.27  0.20  0.61  0.05 | [-0.01:0.65]  [-0,38:0.11]  [-0.51:0.11]  [-0.35:0.21]  [-0.00:0.32] | 0.03  0.01  0.01  0.00  0.03 | | 0.48  0.40 | |
| **Non-intrusiveness**  Sex (girl)  Prenatal distress  Postnatal distress  Current distress  Non-intrusiveness 8 months | 0.36  -0.09  -0.22  -0.08  0.08 | 0.17  0.12  0.16  0.14  0.08 | | 0.03  0.45  0.16  0.55  0.32 | [0.03:0.69]  [-0.34:0.15]  [-0.53:0.09]  [-0.36:0.19]  [-0.08:0.25] | 0.04  0.00  0.02  0.00  0.01 | | 0.60  0.38 | |
| **Non-hostility**  Sex (girl)  Prenatal distress  Postnatal distress  Current distress  Non-hostility 8 months | 0.36  -0.01  -022  -0.09  0.09 | | 0.17  0.12  0.16  0.14  0.08 | 0.03  0.38  0.17  0.54  0.28 | [0.03:0.70]  [-0.35:0.13]  [-0.53:0.09]  [-0.36:0.19]  [-0.07:0.25] | | 0.04  0.01  0.02  0.00  0.01 | | 0.55  0.38 |

**Table 3.** *The associations between mother-infant interaction, maternal psychological distress and child externalizing at 4 years.*

| Variable | *B* | *SE* | | *p* | *CI 95%* | *Partial ETA²* | | *adj p ^a^* | |
| --- | --- | --- | --- | --- | --- | --- | --- | --- | --- |
| **Structuring**  Sex (girl)  Prenatal distress  Postnatal distress  Current distress  Structuring 8 months | -0.15  0.03  0.25  0.20  -0.03 | 0.19  0.17  0.17  0.13  0.10 | | 0.43  0.85  0.14  0.13  0.79 | [-0.54:0.23]  [-0.31:0.38]  [-0.08:0.59]  [-0.06:0.47]  [-0.22:0.17] | 0.01  0.00  0.02  0.03  0.00 | | 0.93  0.38 | |
| **Non-intrusiveness**  Sex (girl)  Prenatal distress  Postnatal distress  Current distress  Non-intrusiveness 8 months | -0.17  0.04  0.24  0.21  0.03 | 0.19  0.18  0.17  0.13  0.10 | | 0.38  0.81  0.16  0.12  0.72 | [-0.54:0.21]  [-0.31:0.39]  [-0.09:0.58]  [-0.06:0.47]  [-0.16:0.23] | 0.01  0.00  0.02  0.03  0.00 | | 0.93  0.38 | |
| **Non-hostility**  Sex (girl)  Prenatal distress  Postnatal distress  Current distress  Non-hostility 8 months | -0.16  0.00  0.27  0.22  -0.12 | | 0.19  0.17  0.17  0.13  0.09 | 0.40  0.99  0.11  0.10  0.22 | [-0.53:0.21]  [-0.34:0.34]  [-0.07:0.60]  [-0.05:0.48]  [-0.30:0.07] | | 0.01  0.00  0.03  0.03  0.00 | | 0.96  0.38 |

**Table 4.** *The associations between mother-infant interaction, maternal psychological distress and child internalizing at 4 years.*

| Variable | *B* | *SE* | | *p* | *CI 95%* | *Partial ETA²* | | *adj p ^a^* | |
| --- | --- | --- | --- | --- | --- | --- | --- | --- | --- |
| **Sensitivity**  Sex (girl)  Prenatal distress  Postnatal distress  Current distress  Sensitivity 8 months | -0.04  0.28  0.03  0.29  -0.02 | 0.18  0.17  0.16  0.13  0.10 | | 0.84  0.10  0.85  0.02  0.85 | [-0.40:0.33]  [-0.05:0.60]  [-0.29:0.35]  [0.04:0.55]  [-0.22:0.18] | 0.00  0.03  0.00  0.05  0.00 | | 0.38  0.93 | |
| **Structuring**  Sex (girl)  Prenatal distress  Postnatal distress  Current distress  Structuring 8 months | -0.04  0.27  0.03  0.29  0.00 | 0.19  0.17  0.16  0.13  0.10 | | 0.82  0.10  0.84  0.02  0.97 | [-0.41:0.33]  [-0.06:0.60]  [-0.29:0.36]  [0.04:0.55]  [-0.19:0.19] | 0.00  0.03  0.00  0.05  0.00 | | 0.38  0.93 | |
| **Non-intrusiveness**  Sex (girl)  Prenatal distress  Postnatal distress  Current distress  Non-intrusiveness 8 months | -0.04  0.29  0.02  0.30  0.04 | 0.18  0.17  0.16  0.13  0.09 | | 0.82  0.09  0.91  0.02  0.66 | [-0.40:0.32]  [-0.05:0.63]  [-0.31:0.35]  [0.04:0.55]  [-0.15:0.23] | 0.00  0.03  0.00  0.06  0.00 | | 0.38  0.95 | |
| **Non-hostility**  Sex (girl)  Prenatal distress  Postnatal distress  Current distress  Non-hostility 8 months | -0.04  0.28  0.03  0.29  0.03 | | 0.18  0.17  0.16  0.13  0.09 | 0.81  0.09  0.85  0.03  0.71 | [-0.40:0.32]  [-0.05:0.61]  [-0.29:0.35]  [0.03:0.54]  [-0.15:0.21] | | 0.00  0.03  0.00  0.05  0.00 | | 0.38  0.93 |

**Table 5.** *The associations between mother-infant interaction, maternal psychological distress and child social-emotional competence at 4 years.*

| Variable | *B* | *SE* | | *p* | *CI 95%* | *Partial ETA²* | | *adj p ^a^* | |
| --- | --- | --- | --- | --- | --- | --- | --- | --- | --- |
| **Sensitivity**  Sex (girl)  Prenatal distress  Postnatal distress  Current distress  Sensitivity 8 months | 0.29  -0.25  -0.09  -0.01  0.10 | 0.20  018  0.18  0.14  0.11 | | 0.14  0.17  0.62  0.93  0.34 | [-0.10:0.69]  [-0.61:0.11]  [-0.44:0.26]  [-0.29:0.26]  [-0.11:0.31] | 0.02  0.02  0.00  0.00  0.00 | | 0.38  0.93 | |
| **Structuring**  Sex (girl)  Prenatal distress  Postnatal distress  Current distress  Structuring 8 months | 0.29  -0.25  -0.09  -0.02  0.08 | 0.20  0.18  0.18  0.14  0.10 | | 0.16  0.17  0.62  0.88  0.47 | [-0.12:0.69]  [-0.61:0.11]  [-0.44:0.26]  [-0.30:0.26]  [-0.13:0.28] | 0.02  0.02  0.00  0.00  0.01 | | 0.38  0.93 | |
| **Non-intrusiveness**  Sex (girl)  Prenatal distress  Postnatal distress  Current distress  Non-intrusiveness 8 months | 0.32  -0.31  -0.04  -0.04  -0.18 | 0.19  0.18  0.18  0.14  0.10 | | 0.11  0.09  0.83  0.79  0.07 | [-0.07:0.70]  [-0.70:0.05]  [-0.39:0.31]  [-0.31:0.24]  [-0.38:0.02] | 0.03  0.03  0.00  0.00  0.03 | | 0.38  0.93 | |
| **Non-hostility**  Sex (girl)  Prenatal distress  Postnatal distress  Current distress  Non-hostility 8 months | 0.32  -0.23  -0,10  -0.02  0.02 | | 0.20  0.18  0.18  0.14  0.10 | 0.11  0.20  0.57  0.91  0.81 | [-0.07:0.71]  [-0.60:0.13]  [-0.45:0.25]  [-0.30:0.26]  [-0.17:0.22] | | 0.03  0.02  0.00  0.00  0.00 | | 0.41  0.93 |

**Table 6.** *Interaction effects of sensitivity, structuring, non-intrusiveness and non-hostility on the associations between maternal prenatal psychological distress and child’s social-emotional problems at 2 years.*

| Variable | *B* | *SE* | *p* | *CI95%* | *Partial Eta²* |
| --- | --- | --- | --- | --- | --- |
| Sex (girl)  Prenatal distress  Postnatal distress  Current distress  Sensitivity x prenatal distress | -0.11  -0.04  0.52  0.03  -0.12 | 0.15  0.11  0.14  0.13  0.07 | 0.49  0.76  0.00  0.83  0.10 | [-0.41:0.20]  [-0.26:0.19]  [0.23:0.81]  [-0.23:0.28]  [-0.26:0.02] | 0.00  0.00  0.10  0.00  0.02 |
| Sex (girl)  Prenatal distress  Postnatal distress  Current distress  Structuring x prenatal distress | -009  -0.01  0.51  0.03  -0.11 | 0.15  0.11  0.14  0.13  0.07 | 0.56  0.91  0.00  0.81  0.10 | [-0.38:0.21]  [-0.23:0.21]  [0.23:0.79]  [-0.22:0.28]  [-0.23:0.02] | 0.00  0.00  0.10  0.00  0.02 |
| Sex (girl)  Prenatal distress  Postnatal distress  Current distress  Non-intrusiveness x prenatal distress | -0.12  -0.03  0.50  0.08  -0.03 | 0.16  0.12  0.15  0.13  0.07 | 0.45  0.83  0.00  0.53  0.57 | [-0.43:0.19]  [-0.27:0.21]  [0.20:0.80]  [-0.18:0.34]  [-0.17:0.09] | 0.00  0.00  0.08  0.00  0.00 |
| Sex (girl)  Prenatal distress  Postnatal distress  Current distress  Non-hostility x prenatal distress | -0.12  -0.05  0.52  0.08  -0.05 | 0.16  0.12  0.15  0.13  0.07 | 0.43  0.67  0.00  0.56  0.45 | [-0.43:0.19]  [-0.30:0.19]  [0.22:0.81]  [-0.19:0.34]  [-0.19:0.09] | 0.01  0.00  0.09  0.00  0.00 |

**Table 7.** *Interaction effects of sensitivity, structuring, non-intrusiveness and non-hostility on the associations between maternal prenatal psychological distress and child’s social-emotional competence at 2 years.*

| Variable | *B* | *SE* | *p* | *CI95%* | *Partial Eta²* |
| --- | --- | --- | --- | --- | --- |
| Sex (girl)  Prenatal distress  Postnatal distress  Current distress  Sensitivity x prenatal distress | 0.33  -0.12  -0.22  -0.05  0.02 | 0.16  0.12  0.15  0.14  0.08 | 0.04  0.33  0.15  0.73  0.83 | [0.01:0.66]  [-0.36:0.12]  [-0.53:0.08]  [-0.32:0.23]  [-0.14:0.17] | 0.03  0.01  0.02  0.00  0.00 |
| Sex (girl)  Prenatal distress  Postnatal distress  Current distress  Structuring x prenatal distress | 0.32  -0.11  -0.21  -0.07  0.09 | 0.17  0.12  0.16  0.14  0.07 | 0.06  0.36  0.18  0.60  0.24 | [-0.01:0.65]  [-0.36:0.13]  [-0.52:0.10]  [-0.35:0.20]  [-0.06:0.23] | 0.03  0.01  0.02  0.00  0.01 |
| Sex (girl)  Prenatal distress  Postnatal distress  Current distress  Non-intrusiveness x prenatal distress | 0.36  -0.08  -0.23  -0.08  0.02 | 0.17  0.13  0.16  0.14  0.07 | 0.04  0.54  0.16  0.56  0.76 | [0.02:0.69]  [-0.34:0.18]  [-0.55:0.09]  [-0.36:0.20]  [-0.12:0.16] | 0.04  0.00  0.02  0.00  0.00 |
| Sex (girl)  Prenatal distress  Postnatal distress  Current distress  Non-hostility x prenatal distress | 0.35  -0.07  -0.23  -0.10  0.06 | 0.17  0.13  0.16  0.14  0.07 | 0.04  0.62  0.14  0.47  0.40 | [0.02:0.69]  [-0.33:0.20]  [-0.55:0.08]  [-0.39:0.18]  [-0.09:0.21] | 0.04  0.00  0.02  0.00  0.01 |

**Table 8.** *Interaction effects of sensitivity, structuring, non-intrusiveness and non-hostility on the associations between maternal prenatal psychological distress and child’s internalizing and externalizing symptoms at 4 years.*

| Variable | *B* | *SE* | *p* | *CI95%* | *Partial Eta²* |
| --- | --- | --- | --- | --- | --- |
| **Non-intrusiveness on internalizing**  Sex (girl)  Prenatal distress  Postnatal distress  Current distress  Non-intrusiveness x prenatal distress | -0.03  0.11  0.11  0.31  -0.16 | 0.18  0.20  0.17  0.13  0.09 | 0.88  0.60  0.53  0.02  0.08 | [-0.38:0.33]  [-0.29:0.50]  [-0.23:0.45]  [0.06:0.57]  [-0.34:0.02] | 0.00  0.00  0.00  0.06  0.03 |
| **Structuring on externalizing**  Sex (girl)  Prenatal distress  Postnatal distress  Current distress  Structuring x prenatal distress | -0.12  -0.07  0.31  0.22  -0.16 | 0.19  0.19  0.17  0.13  0.11 | 0.53  0.70  0.08  0.11  0.15 | [-0.50:0.26]  [-0.43:0.30]  [-0.03:0.65]  [-0.05:0.48]  [-0.37:0.06] | 0.00  0.00  0.03  0.03  0.02 |
| **Non-intrusiveness on externalizing**  Sex (girl)  Prenatal distress  Postnatal distress  Current distress  Non-intrusiveness x prenatal distress | -0.16  -0.10  0.31  0.22  -0.12 | 0.19  0.21  0.18  0.13  0.10 | 0.41  0.64  0.08  0.10  0.21 | [-0.53:0.22]  [-0.51:0.31]  [-0.04:0.67]  [-0.05:0.48]  [-0.31:0.07] | 0.01  0.00  0.03  0.03  0.02 |
| **Non-hostility on externalizing**  Sex (girl)  Prenatal distress  Postnatal distress  Current distress  Non-hostility x prenatal distress | -0.14  -0.12  0.32  0.24  -0.10 | 0.19  0.20  0.17  0.13  0.09 | 0.44  0.57  0.07  0.07  0.28 | [-0.51:0.23]  [-0.52:0.29]  [-0.02:0.67]  [-0.02:0.51]  [-0.28:0.08] | 0.01  0.00  0.04  0.04  0.01 |

**Table 9.** *Interaction effects of sensitivity, structuring, non-intrusiveness and non-hostility on the associations between maternal prenatal psychological distress and child’s social competence at 4 years.*

| Variable | *B* | *SE* | *p* | *CI95%* | *Partial Eta²* |
| --- | --- | --- | --- | --- | --- |
| Sex (girl)  Prenatal distress  Postnatal distress  Current distress  Sensitivity x prenatal distress | 0.29  -0.24  -0.09  -0.01  0.01 | 0.20  0.19  0.18  0.14  0.13 | 0.15  0.19  0.62  0.93  0.93 | [-0.11:0.69]  [-0.62:0.13]  [-0.44:0.26]  [-0.29:0.27]  [-0.24:0.26] | 0.02  0.02  0.00  0.00  0.00 |
| Sex (girl)  Prenatal distress  Postnatal distress  Current distress  Structuring x prenatal distress | 0.31  -0.31  -0.06  -0.01  -0.10 | 0.20  0.20  0.18  0.14  0.11 | 0.14  0.11  0.76  0.93  0.40 | [-0.10:0.71]  [-0.70:0.08]  [-0.42:0.30]  [-0.29:0.27]  [-0.32:0.13] | 0.02  0.03  0.00  0.00  0.01 |
| Sex (girl)  Prenatal distress  Postnatal distress  Current distress  Non-intrusiveness x prenatal distress | 0.32  -0.33  -0.03  -0.04  -0.02 | 0.20  0.22  0.19  0.14  0.10 | 0.11  0.13  0.88  0.80  0.87 | [-0.07:0.71]  [-0.76:0.10]  [-0.40:0.34]  [-0,31:0.24]  [-0.22:0.18] | 0.03  0.02  0.00  0.00  0.00 |
| Sex (girl)  Prenatal distress  Postnatal distress  Current distress  Non-hostility x prenatal distress | 0.32  -0.28  -0.08  -0.01  -0.04 | 0.20  0.22  0.19  0.14  0.10 | 0.11  0.21  0.67  0.96  0.71 | [-0.07:0.72]  [-0.71:0.16]  [-0.45:0.29]  [-0.29:0.28]  [-0.23:0.16] | 0.03  0.02  0.00  0.00  0.00 |
